# Perceptions of Local Environmental Issues and the Relevance of Climate Change in Nepal's Terai: Perspectives From Two Communities

**DOI:** 10.3389/fsoc.2019.00060

**Published:** 2019-08-20

**Authors:** Nick Nash, Stuart Capstick, Lorraine Whitmarsh, Indra Chaudhary, Rija Manandhar

**Affiliations:** ^1^Tyndall Centre for Climate Change Research, School of Psychology, Cardiff University, Cardiff, United Kingdom; ^2^Research Department, Institute for Social and Environmental Research Nepal (ISER-N), Bharatpur, Nepal

**Keywords:** climate change, environment, perception, community, local, Nepal, culture

## Abstract

The direct and indirect impacts of global climate change entail serious consequences for global biophysical and social systems, including the health, well-being and sustainability of communities. These impacts are especially serious for vulnerable groups in economically developing societies. While climate change is a global phenomenon, it is at the local level that impacts are most felt, and from where responses to climate change are enacted. It is increasingly urgent that communities possess the capacity to respond to climate change, now and in the future. Community representations of climate-relevant issues are critical to underpinning responses. Environmental representations do not directly reflect actual physical conditions but are interpreted through social and cultural layers of understanding that shape environmental issues. This paper investigates environmental and climate-relevant perceptions within two communities in the Terai region of Nepal; the city of Bharatpur and the village of Kumroj in Chitwan Province. Following mixed findings on levels of climate change awareness in Nepal, we set out to explore perspectives on the environment and climate change awareness by conducting 30 qualitative interviews with local people. The study found that issues linked to sanitation and cleanliness were most important in both communities, while reports of temperature and weather changes were less common and typically linked to local causes rather than climate change. Imagined futures were also closely related to current environmental issues affecting communities and did not discuss climate change, though temperature and weather changes were anticipated. However, when talk of climate change was deliberately elicited, participants displayed their awareness, though this was rarely linked to local conditions. We conclude that, in light of other pressing local issues, climate change is yet to penetrate the environmental representations of some communities and there is a need to address the disconnect between local issues and global climate change. Making climate change relevant at the local level by connecting to salient local issues and co-benefits comprises an important step in bridging the gap between more global awareness and its relevance more locally, particularly for communities at risk.

## Introduction

Climate change impacts are set to profoundly change global ecological and social systems, bringing about fundamental changes to human behavior (Evans, 2019). The complexity of global climate systems makes it difficult to accurately predict the nature of climate change impacts, though a degree of certainty rests in knowing that fundamental lifestyle shifts commensurate with the scale of climate change will be required if we are to limit the global temperature increase to 1.5°C by 2100 (Rogelj et al., 2018). In addition to average temperature increase, societies also face increases in the frequency of extreme weather events, air pollution and sea level rise, posing an array of physical threats to human health and well-being, both directly and indirectly (Watts et al., 2018).

Consequently, the impacts of sudden natural disasters (such as shock, emotional distress and post-traumatic stress), and cumulative stresses over time (for example, changes to livelihoods, economic opportunities and social support) from climate change carry serious psychological impacts for those affected (Clayton et al., 2015). These impacts are especially pronounced for citizens living in economically developing countries, particularly for those within developing countries who rely on natural resources to sustain their livelihoods (Aryal et al., 2014).

In addition to continued mitigation, societies will be required to adapt to current and future environmental change. Adaptation in this context refers to a community's capacity to deal with changes, reduce vulnerability to risks, and improve the well-being of communities (Bhatta et al., 2015). While action on climate change maintains a crucial global imperative (Gupta, 2010), variability in environmental impacts and sociocultural differences at the local level also highlight the need to better understand the contexts within which responses to climate-relevant issues occur (Adger, 2003). While global environmental issues such climate change are constructed in top–down ways through scientific, political and other cultural narratives (Adger et al., 2013), they are also blended with and filtered through more vernacular, localized forms of understanding (Byg and Salick, 2009).

In this paper we investigate environmental and climate-relevant perceptions in the context of two rural communities in the Terai (lowland) region of Nepal. Nepal is an economically developing country in South Asia that faces serious impacts from climate change including a predicted temperature increase of 2.8°C by 2060 and up to 4°C by 2090, snowpack melt, glacier retreat, shifting climatic zones, increased extreme weather events, increased periods of drought and erratic precipitation (Becken et al., 2013). In a country where agriculture is the principle industry for 80% of citizens (Paudel et al., 2019) and widespread poverty exists, many of Nepal's citizens are precariously positioned by climate change threats (Leichenko and Silva, 2014).

Following Smit and Wandel (2006), we take a bottom-up approach to environmental and climate-relevant perceptions at the community level. We discuss the findings from 30 qualitative interviews with community members, focusing on the role of subjective environmental perceptions relating to current and future environmental issues, including community perspectives on climate change, with a focus on the impacts for human well-being. While scientific measurement of ecological impacts provides the foundation for mitigation and adaptation, community perceptions are also critical to ensuring that policy interventions fit community understandings and avoid being misinterpreted or rejected by the community (Leiserowitz, 2007). The Inter-Governmental Panel on Climate Change (IPCC) has also stipulated that local knowledge should be used to inform climate adaptation planning (Carter, 2019).

In addition to comprising physical phenomena, environmental issues, including climate change, comprise important social, cultural, and political dimensions that mediate perceptions of the physical (Hulme, 2009; Whitmarsh, 2011). These are both facilitated and constrained by cultural knowledge, expressed through social norms, practices, institutional structures and prescribed roles and ways of living. The extent to which climate-relevant communications, interventions and policy are received, understood and enacted by local communities therefore depends on the degree to which top-down standardized scientific narratives converge with, or diverge from the micro-contexts of localized forms of knowledge (Zinn, 2004). Culturally-filtered observations and experiences of environmental conditions are a crucial way in which citizens understand environmental conditions and processes of change (Bickerstaff, 2004; Hulme, 2012). Human cognitive biases also influence and distort environmental perceptions. For example, more unusual or memorable weather events tend to exert a stronger influence on perceptions (Trenberth et al., 2015).

Furthermore, perspectives of global climate change may be constrained due to being beyond human perceptual capacity. This means that other locally-salient issues may be perceived as more immediate (Weber, 2010). While people may attribute extreme weather events to global climate change, such interpretations depend on culturally-available narratives that construct such issues, whereas physical climate change is, arguably, only discernible over long time periods. Essentially, a single event cannot unequivocally be attributed directly to climate change, though an individual may or may interpret it as such, depending on their perspective (Hulme, 2014). Similarly, interpretations of local environmental conditions have been found to influence more global climate-relevant understandings. For example, in one study, local perceptions of deforestation, urbanization and air pollution framed explanations of climate change (Maharjan and Joshi, 2012). This suggests that people look for proximate and visible causes in the absence of wider understanding.

Nonetheless, studies have demonstrated evidence that communities who are more in touch with their surroundings are able to accurately detect environmental changes, such as seasonal temperature and weather fluctuations (Gurung, 1989; Tiwari et al., 2010; Poudel and Duex, 2017; Uprety et al., 2017). Other research has found that while community members are accurate in their perceptions of some seasonal and weather-related changes, they are less accurate at perceiving others (Myers et al., 2013). Environmental impacts also affect different groups within a country or region differently, and not always uniformly (Gentle et al., 2014) and may even be experienced differently by different members of the same community (Maharjan and Joshi, 2012).

Climate change awareness has been reported to be higher in economically developed countries than in economically developing nations, a pattern also found for countries within Asia (Maharjan and Joshi, 2012). Other research has found educational attainment to be the strongest predictor of awareness (Lee et al., 2015). Cultural differences are also evident in terms of climate change risk perceptions; in Latin America and Europe, comprehension of the anthropogenic origin of climate change has been found to be the strongest predictor, while in several Asian and African countries, perception of temperature increase locally was most influential (Lee et al., 2015). Perceptions of temperature and weather change are widespread. Savo et al. (2016) conducted a meta-analysis of 10,660 change observations reported across 2,230 localities in 137 countries, which showed increases in temperature, and changes in seasons and rainfall patterns in 70% of localities in 122 countries.

Nepal is particularly susceptible to climate change, with change in the Himalaya accelerating beyond the global average (Zomer et al., 2014). In the Terai agriculture is the principle economic activity, with around 80% of the population dependent on farming for their livelihoods. Therefore, climate change carries significant risks for the economy, which indirectly affect food production and security. The situation is exacerbated by widespread poverty; in 2010 over 25% of the population subsisted below the national poverty line (Adhikari, 2018). Poorer groups within society are more likely to be exposed to climate stresses and possess fewer resources to adapt (Gentle et al., 2014; Leichenko and Silva, 2014). Nepal is divided into three ecological regions comprising the *Terai* (lowland), *hill* and *mountain* regions, each of which is characterized by different ecological and climatic conditions. The Terai forms a fertile plain located in the south of the country where the majority of food production takes place, and is also the most densely populated region (Paudel, 2012). Of relevance within Nepal, food shortages due to seasonal changes, infestations of new crop pests and a decline in soil productivity have been recorded (Paudel, 2012).

Public awareness is seen as a major limitation to climate change adaptation within Nepal (Withana and Auch, 2014). While some studies have found high levels of climate change awareness amongst Nepalese citizens (Becken et al., 2013), other research has found awareness to be low (Gallup, 2009). In a cross-national study of 5,060 households, Tanner et al. (2018) report that climate change awareness was low (<50% were aware of the phenomenon even if they had been aware of changes in the weather). Awareness in urban areas was lower than in rural areas (56% v 46%), and very low in mountain areas (63% had not heard of climate change). There were also significant proportions of citizens who did not perceive that the climate was changing. Maharjan and Joshi (2012) report that among the Chepang community only 11.8% of respondents had heard of climate change; of those, only 4.8% were able to relate the phenomenon to changes in weather patterns, temperature, rainfall, wind, floods, landslides, and environmental change.

Research on community perceptions of environmental and climate-relevant change in Nepal has recorded perceptions of warmer summers (Tiwari et al., 2010; Uprety et al., 2017); milder winters (Dahal, 2005; Maharjan and Joshi, 2012; Becken et al., 2013); more erratic rainfall (Chapagain et al., 2009; Paudel, 2012; Becken et al., 2013; Devkota and Bhattarai, 2018); increased periods of drought (Tanner et al., 2018); and more frequent foggy days (Shrestha et al., 2018). However, community perceptions are not consensual. Maharjan and Joshi (2012) report that while 47.5% believed that summers were getting warmer, nearly 10% reported that summers were becoming cooler and 38% perceived no change. In addition, 21% believed that winters were getting colder while 22% believed that winters were becoming milder. Furthermore, 37% believed that there was less rain overall, while 13–17% perceived no change in rainfall. They attribute this to differences in “visual salience”; whereby rainfall is more conspicuous and facilitates perception, whereas temperature change is less directly observable.

With specific reference to the Terai region, Maharjan et al. (2011) interviewed farmers in the Western Terai, with 90% of respondents reporting increases in climate-related risks (erratic rainfall, flooding, droughts, riverbank erosion, windstorms, hailstorms, insect infestations). Tiwari et al. (2010) surveyed Terai communities in which over 75% of participants reported delayed onset of the monsoon and changes in flowering and fruiting time for some plant species. Meanwhile, Manandhar et al. (2011) found that more than two-thirds of farmers in the Terai claimed to have personally experienced evidence of climatic change.

As a result of perceived environmental change in the Terai, and in other regions livelihoods and lifestyles are adapting to changing conditions. Khanal et al. (2018) surveyed farming households in Nepal to gauge adaptation practices across the three ecological regions of Nepal, reporting that 91% of households had adopted at least one practice to minimize impacts of climate change. Adaptation may be more anticipatory or reactive and distinguished by duration, scale of implementation (i.e., more local or more widespread) and focus (e.g., behavioral, institutional, economic, technological, informational) (Smit et al., 2000). In a study of climate change adaptation in the rural hill region of Nepal, Gentle et al. (2018) examined household responses in four villages. Adaptive responses to climate change in rural communities were found to be less coordinated and more reactive and unplanned rather than anticipated and coordinated.

Changes to agricultural practices constitute a primary focal point for adaptation and change. These have included changes in the times crops are sown and harvested (Maharjan et al., 2011), switching to more climate resilient crop varieties and tree and plant species (Maharjan et al., 2011; Paudel, 2016; Gahatraj et al., 2018), as well as increased use of pesticides, and income diversification (Gentle et al., 2018). Climate change is also perceived as benefiting some crop species (Rawal and Bharti, 2015). For example, mangos are being grown at higher altitudes than was possible in the past (Chapagain et al., 2009).

Within villages, water practices were changing to conserve water resources (Tiwari et al., 2010), and changes to diets have also been identified (Tanner et al., 2018), with less rice being consumed due to the effects of climate on rice productivity (Maharjan and Joshi, 2013). Two-story houses are increasingly being constructed for food storage and as refuge from flooding (Maharjan and Joshi, 2013), while buildings are being oriented to withstand windstorms, incorporating single rather than double doors (Maharjan and Joshi, 2013). Seasonal migration and resettlement becoming more common (Prasain, 2018). People are also reported to be planting more trees and grasses on their own land as well as on communal land to protect communities from flooding, wind and dust (Tiwari et al., 2010; Maharjan et al., 2017). Withana and Auch (2014) report that afforestation is viewed as the most effective climate change adaptation strategy by communities.

In summary, perceptions of environmental conditions are key to informing behavior, including the need to adapt to a changing climate. In the context of Nepal, adaptation is particularly salient and it is critical that communities respond to environmental risks in ways that ensure the well-being and futurity of those communities. Given that studies of climate-relevant perceptions have reported mixed findings in terms of awareness, we seek to clarify how Nepalese communities view environmental issues now and in the future. Such perceptions act as important indicators of how local communities make sense of what is happening in their surroundings.

Following our review of the literature, the following questions guide the study approach:

- How do communities in Nepal's Terai perceive their environment?
- How do they see that environment changing in the future?
- To what degree are local communities aware of climate change?
- What is the relative importance of climate change compared to other issues environmental affecting the community?

## Materials and Methods

The following subsections describe the study design and procedure. Broadly, this comprised a qualitative approach using semi-structured interviews with residents in two communities in the Terai region of Nepal. Thirty interviews were conducted in total. 15 interviews were conducted with residents of the village of Kumroj, a small rural community bordering Chitwan National Park. Another 15 interviews were conducted with residents of Bharatpur, an urban community approximately 12 miles (20 km) away. For each group, we were interested in gauging perceptions of salient environmental issues, including climate change. We selected two different communities to explore the degree to which locally salient issues varied and informed discussions. Before commencing fieldwork, the study design was scrutinized and approved by the Research Ethics Committee in the School of Psychology at Cardiff University.

### Participants

Fieldwork was conducted in January and February 2016. A purposive sampling strategy (Silverman, 2015) was used to try to generate a range of different sociodemographic profiles within each community in terms of age, gender and ethnicity. All participants were aged 18+ and resided in either Bharatpur or Kumroj, both in the Chitwan district. Bharatpur has a population of 280,000 and is one of the largest and fastest growing cities in Nepal. While it is home to a number of small-scale processing industries, agriculture remains the biggest industry. Kumroj is a small town with a population of 8,000. Kumroj borders Chitwan National Park, the first National Park created in Nepal (in 1973). In recent years in-migration has increased pressure on land for settlement and agriculture. Increasing tourism has put additional pressure on the landscape. A number of community development initiatives have attempted to confer Kumroj as an ecological exemplar, with the creation of a community forest initiative and grant funding to encourage domestic biogas installation to reduce deforestation, launched on World Environment Day, 2013. Around 80% of households within Kumroj have installed bio-gas converters to reduce reliance on the forest for fuel.

To arrange fieldwork with local people in Kumroj, we contacted the offices of the World Wildlife Fund for Nature (WWF) in Kathmandu, who had been involved in community development projects in Kumroj. Through WWF, we were able to negotiate access through local community leaders who helped us to recruit participants. Prior to our arrival, the study was advertised by word-of-mouth by community leaders, who identified potential members of the community willing to be interviewed. Extra care had to be taken in gaining access to participants, establishing contact and opening communicative spaces with the community, which could be damaged if pushed too quickly (Wicks and Reason, 2009). The study was promoted as a “*lifestyle and behavior*” project and avoided making reference to the environment, as we wished to avoid recruiting only those members of the community whose motivations and values were strongly pro-environmental. At recruitment, a brief screening procedure was applied; individuals were screened to ensure that they were 18+ and aware of the broad purpose of the study and what would be required in terms of participation. We also purposefully recruited individuals to ensure that we had a roughly equal split in terms of gender, as well as diversity in terms of age, ethnicity, occupation, and income. See Table 1 for subsample demographics.

**Table 1:** Subsample demographics.

		**All** **(*n* = 30)**		**Bharatpur** **(*n* = 15)**		**Kumroj** **(*n* = 15)**	
*Gender*	*Female*	13	43.3%	7	46.7%	6	60%
	*Male*	17	56.7%	8	53.3%	9	40%
*Age group*	*18–24*	6	20%	3	20%	3	20%
	*25–34*	8	26.7%	6	40%	2	13.3%
	*35–44*	5	16.7%	2	13.3%	3	20%
	*45–54*	6	20%	2	13.3%	4	26.7%
	*55–64*	0	0%	0	0%	0	0%
	*65+*	3	10%	2	13.3%	1	6.7%
	*Not stated*	2	6.6%	0	0%	2	13.3%
*Household income per annum*	*Re −10,000*	1	3.3%	0	0%	1	6.7%
	*Re 10,000–19,999*	0	0%	0	0%	0	0%
	*Re 20,000–29,999*	2	6.7%	2	13.3%	0	0%
	*Re 30,000–39,999*	0	0%	0	0%	0	0%
	*Re 40,000–49,999*	3	10%	1	6.7%	2	13.3%
	*Re 50,000+*	23	76.7%	12	80%	11	73.3%
	*Not stated*	1	3.3%	0	0%	1	6.7%

To recruit our Bharatpur subsample, we collaborated with the Institute for Social and Environmental Research Nepal (ISER-N). ISER-N is a research and development institute that conducts applied research to inform policy-making and effective sustainable development initiatives across local communities. Using a similar method to the above, ISER-N acted as our guide and point of access to the local community and advertised and recruited a subsample of local people who had expressed an interest in discussing their lifestyles and behaviors.

### Procedure

Once participants had been identified, screened, and given further information about the study, they were invited to take part in an interview to discuss aspects of their day-to-day lifestyles and behaviors with the research team. Interviews were scheduled to take approximately one-and-a-half hours, but varied from 45 min to 2 h. A semi-structured interview method (Galletta, 2013) was chosen in which a standard set of questions was covered while also allowing flexibility for follow-up questions and exploration of other issues of relevance to participants. Such flexibility is an advantage in cross-cultural settings as this allows for greater exploration of cultural factors underpinning issues of interest (McIntosh and Morse, 2015; Hagaman and Wutich, 2017). All participants were required to give written informed consent prior to participation.

Questions in the interview protocol sought to contextualize environmental perspectives within people's wider everyday lives as far as possible. Questions broadly covered perceptions of the environment and the importance of environmental issues environmental problems (including climate change), engagement in environmentally-friendly behavior, the character, motivations for and consequences of behaviors, and comparisons with others in terms of acting in environmentally-friendly ways (see Supplementary Information).

The majority of interviews took place at participants' homes. Discussions took place on seats or woven mats in the front yards of houses rather than inside the building itself. A small number of interviews were conducted in other locations, such as a local café, or community building in the case where the home could not be used. We relied heavily on our collaborators and local community leaders to manage interview arrangements in line with our concerns about accessing members of an unfamiliar culture and wishing not to transgress social boundaries. Because people's yards are the area of the home where a lot of day-to-day interaction takes place, providing socially appropriate spaces for interaction.

One of the disadvantages of holding interviews outside was that on some occasions the research team's presence would attract the curiosity of other family members, neighbors and other locals. The sudden presence of others could occasionally alter the dynamic of the interview interaction, particularly if the others who were present began talking or offering their own perspectives. On one or two occasions the research team had to ask bystanders to limit their contribution so as to allow the participant to speak. To a cultural outsider this would appear potentially problematic and non-conducive to an appropriate interview context, which led us to consider this and other ethical considerations in conducting interview research in different cultures.

#### Ethical Considerations in Conducting Interviews in Different Cultures

Researchers typically assume that the communities in which they work will be aware of the concept of research and its value, though for many communities research is something abstract, distanced and difficult to make sense of in relation to their ordinary lived experience. This came across clearly in working with each subsample. In Bharatpur, participants were familiar with ISER-N and, owing to participating in other cross-cultural research, were more comfortable with the researcher's presence than participants in Kumroj, who had not been so exposed to researchers and the research process. Further to this, bridging communicative spaces is not confined merely to issues of translation and word equivalence, but of more conceptual differences in terms of the ways that different cultures define reality and categorize their experience (Fong, 2012). Language and culture are woven together in ways that require not only the translation of speech, but the translation of cultural meanings that are often concealed from those outside of that culture. In designing the interview protocol, we worked closely with our collaborators not only to ensure that questions were understandable, but that any cultural assumptions (for example, about the lifestyles, values, and practices of the community) were identified and addressed appropriately. All interview materials were double-translated.

Qualitative methods including interviews, also carry particular ethical implications in terms of power imbalance, where the discussion is primarily directed by the researcher (King et al., 2018). Assumptions about the identity of the western researcher (typically white, middle-class, and educated) on the part of the research participant construct interactional dynamics before a word has been spoken. Similarly, the reflexive researcher will not only consider how their own identity might influence communication, but how their assumptions about the community they are researching enter into framing interactions. While researchers may seek to embark on research practices that are non-exploitative and non-oppressive, researchers are nonetheless complicit in systems of oppression and should be aware of their own privileges.

The interview team comprised a male researcher (lead author) and a female translator to minimize any gender imbalance that might affect trust and participant disclosure, especially for female participants (Campbell and Wasco, 2000; Sikes, 2018). The translator also played an active role in facilitating each interview, asking additional questions and clarifying understanding, as opposed to simply translating questions and responses. It was felt that a combination of cultural insider and cultural outsider benefited the discussion; while the former helped to increase trust and disclosure, the latter encouraged more detailed exploration of issues that might otherwise be taken for granted by those familiar with those issues (Dwyer and Buckle, 2009).

Conducting qualitative fieldwork in collaboration with translators can also compromise the quality and accuracy of the material generated. In an interview context, the translator adds an additional layer to the interaction. For example, the translator is likely to be more acquainted with the cultural nuances of the interaction than the researcher. Therefore, both the researcher and translator can affect the fieldwork process, as well as disrupting the flow of talk to allow for translation (Van Teijlingen et al., 2011). When fully transcribed, interviews can also show disparity between participant responses and translated responses. van Teijlingen et al. suggest that a way round this is to allow the translator to conduct the interview and only relay main points to the researcher, though this can be impractical, as well as excluding the researcher.

Prior to the interviews, considerable time was spent in developing and pre-testing interview questions. After constructing an initial set of interview questions in English, these were double-translated and then reviewed by our collaborators in Nepal. This was invaluable in not only identifying significant weaknesses in conceptual equivalence between Nepali and English versions of the questions (Larkin et al., 2007), but also in highlighting researchers' cultural assumptions inherent in questions relating to everyday life in “other” places. That is, while a translated question may be conceptually equivalent to the original, it still may not be understandable in another culture (e.g., where researchers from one culture assume that all participants in another culture will have the same access to resources, such as running water). Even when all care is taken with translation, translators may be unfamiliar with a particular geographical region or cultural group. Therefore, it is recommended that questions are pre-tested in the specific cultural contexts in which they will be used.

With reference to interview locations, our decision to hold interviews outside and not in a more private location was primarily guided by social conventions as well as pragmatism, though we acknowledge the active influence of the nature of the space within which such interactions take place (Gagnon et al., 2015). As mentioned at the end of the previous section, on occasion others were present at interviews in ways that influenced participant responses and could have constrained disclosure or breached informal assumptions about confidentiality (though interview questions were not considered to cover personally sensitive topics). Conversely, in discussing lifestyle and behavior issues, the home sometimes served as an exemplar in which participants described their activities in the context of the physical surroundings, which enhanced disclosure. In addition, conducting interviews in familiar environments can reduce the power imbalance between researcher and research participant (Gagnon et al., 2015).

Ethical considerations do not end at the point at which the interview concludes but influence ongoing reflections following the interview (such as translation, analysis, writing-up and dissemination) (Hoover et al., 2018). Acknowledging that translation imposes an additional level of interpretation on the spoken word (Caretta, 2015), we have tried insofar as possible to contextualize accounts based on participants' direct speech rather than translators' interpretations of what was said. At the end of each interview, participants were provided with a verbal and written debrief in Nepali, in which they were given the opportunity to get in touch with the research team through appointed members of the local community and in-country collaborators should they have any further questions or concerns once participation had ended.

#### Analytic Approach

All interviews were digitally audio-recorded and translated and transcribed at ISER-N. Written field notes were also taken throughout each interview relating to points of interest and things that might not be captured by the recorder. An “*in-interview*” system of translation was used whereby questions and responses were translated between English and Nepali by the translator. This method of translation was primarily used to aid communication within the interview itself. When the interview recordings were translated, the translators re-translated participants' responses, which appear alongside the in-interview translations in the transcripts. This was done as the task of translating what at times were lengthy utterances in the moment, could have led to omissions and gaps, whereas in translating participant responses from the recordings utterances could be replayed and listened to repeatedly for clarity, thus better capturing what was said.

An episodic narrative approach was used as an analytic framework by which to explore participants' accounts of environmentally-friendly perceptions and behaviors. This approach treats perceptions and experiences as lived narratives situated within the wider society and culture (Flick, 2000; Jovchelovitch and Bauer, 2000). Narrative interviewing is interested in eliciting particular episodes or features of participants' lives and how they make sense of the world as embodied, culturally and spatially situated individuals (Raulet-Croset and Borzeix, 2014). Interview audio files and transcripts were analyzed using NVivo 11, supplemented by written field notes.

## Analysis

Our analysis combined several methods, which we outline here. In line with the early stages of a grounded theory approach (Timmermans and Tavory, 2012) we began by reading through transcripts to identify examples of talk that involved themes relating to health and well-being in the context of environmental issues. As much as possible, given inevitable researcher preconceptions and positions (Caelli et al., 2003) we sought to identify general themes and provisional topics of interest, without imposing a predetermined framework. This manner of bottom-up or inductive analytic reading of the data allows for the broadest possible range of salient themes to be identified. Once we familiarized ourselves with the material through repeated readings, we then developed a system of codes to more precisely categorize these themes. In order to do so, we used a version of template analysis, which is suitable for identifying themes in research data that is commensurate with both essentialist and constructionist perspectives, and which enables a hybrid approach that utilizes both inductive and deductive techniques (Brooks et al., 2015). Template analysis is a form of thematic analysis applied to qualitative data, that is sensitive both to emergent themes that are grounded in the data (i.e., not anticipated or predetermined by the researcher) as well as permitting predetermined codes or categories to be applied (i.e., in line with the researcher's interests and the existing literature). The coding framework was developed through an iterative process: through multiple readings of the research data and refinement of initial codes until further changes to the framework did not enhance it further. A further feature of template analysis is the development and application of a hierarchical coding approach, designed to shed light on the structure across the set of codes. In the case of the present study, this for example has led to higher-level codes such as “behavioral responses” beneath which we identify sub-codes such as “cleanliness” and “waste disposal.”

In the analysis that follows, we present extracts from interviews in both communities comprising perceptions of environmental issues. Where considering the themes identified within the data, we have illustrated this using a single typical extract and alluded to its occurrence in other participants' accounts within the text.

### Community Perceptions of Current Environmental Conditions

**Figure 1:**
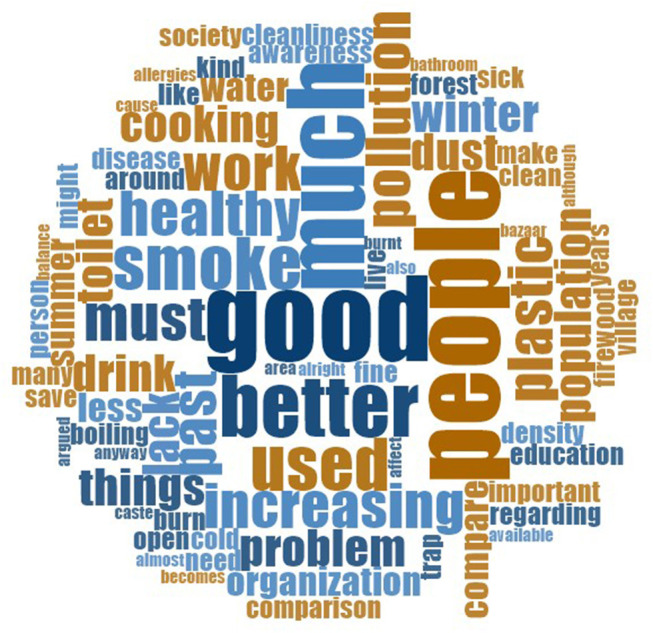
Word cloud of the 75 most common words associated with present environmental conditions (Bharatpur participants).

**Figure 2:**
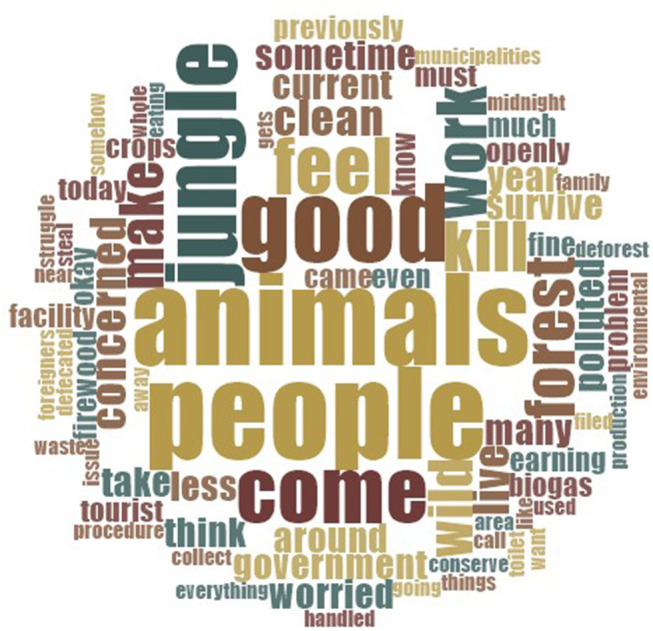
Word cloud of the 75 most common words associated with present environmental conditions (Kumroj participants).

We began by asking participants about the importance of environmental issues in their day-to-day lives and what the surrounding environment was like. Responses comprised both positive and negative evaluations of environmental conditions, though there was a greater range of issues forming the latter. To get some sense of the kinds of terms used to describe the local environment in each community, we created two word clouds using NVivo, which display the most frequently used words in discussing issues. The results of these are displayed in Figures 1, 2.

Both communities used the same terms in discussing the environment, such as “*people*” and “*good*.” In Bharatpur, “*better*” was also commonly used in talking about the environment, which may reflect the dominance of the issue of sanitation (see section Sanitation and Hygiene below). Negative words such as “*pollution,”* “*smoke,”* “*problem,”* and “*dust”* also came up relatively regularly, as did the word “*plastic*.” Terms relating to hygiene and sanitation were also notable. These included “*cleanliness,”* “*toilet*,” “*healthy,”* and “*clean*.” Meanwhile, in Kumroj, commonly used terms appeared congruent with the community's rural position. These included “*animals*,” “*jungle*,” “*wild*,” and “*forest*.” Words such as “*polluted*,” “*concerned,”* and “*worried”* were also used. We now move on to discuss responses in more detail.

#### Sanitation and Hygiene

The primary way issue through which the environment was assessed in both communities, though particularly in Bharatpur, related to sanitation and the need to maintain a clean environment to reduce the risks of disease:

“*Previously, like ten to fifteen years ago people used to smoke, and there was open defecation everywhere, there weren't any toilets, so people used to get sick and the death rate also used to be very high, people used to be suffering by many kinds of disease, skin problems, allergies. Now currently almost every household has a toilet, and many organizations have been working on cleanliness. They have been providing various training and awareness programs regarding the clean environment. So now I would say, the environment is not so bad around here*.” (Bharatpur, Interview A3).

In the above account, a positive assessment of environmental conditions is formulated by drawing a comparison between past and present sanitation and sanitary practices. Whereas, in the past, communities were affected by diseases resulting from unsanitary conditions, this had now changed, providing a positive indicator of the local environment as a whole.

In addition to health risks from open defecation, providing proper toilets in rural communities such as Kumroj also minimized other risks from wildlife, and the discomfort of adverse weather conditions:

“*If we don't have a toilet, then we may have to face many difficulties such as while going outside for toilet then we might get attacked from snake or when raining it would be hard to go the toilet. And if we openly defecate then it will pollute the environment and as result we may have to suffer from different diseases, so environment is the most important thing to survive for everyone and we can't imagine life without environment*.” Kumroj, Interview B11).

For rural communities, development of sanitation was considered not only key to well-being, but also, implicitly, key to a *good* environment. Talk of sanitation in the context of evaluating the local environment also rested heavily on community awareness. What contributed to a lack of environmental quality in the past was not only that proper sanitation was unavailable, but that in the past, communities were less aware of the risks to health and well-being from poor sanitation. Risky sanitary practices were thus maintained as people did not know any better. In contrast, nowadays, communities were more aware of risks from inadequate sanitation and knew how to overcome issues such as contaminated drinking water. In this way, community awareness also contributed to positive judgements of environmental quality:

“*The* e*nvironment here is better in comparison to the past…These facilities didn't exist. There had been problems of drinking water taps. The same tap was used. It wasn't enough. In society, people had to drink water from wells. They had germs, smoke and dust*.” (Bharatpur, Interview A10).

Similar to the accounts of the shift to a better environment through the development of toilet facilities and reduction in the practice of open defecation, an overall positive evaluation of *the* environment is constructed through comparisons of past and present. For many participants, issues of health, sanitation and hygiene formed the yardstick by which the overall environment was evaluated positively.

#### Waste and Pollution

While improvements in sanitation and hygiene across both communities provided a positive indicator of environmental quality, there was more ambivalence where participants discussed other issues indicative of environmental quality in their respective communities. For participants in both Bharatpur and Kumroj, distance from industrial development and proximity to green spaces were important factors associated with positive environmental assessments:

“*The environment around here is ok, there is no industry and factory so it is not that much polluted here and we are nearby jungle so we have greenery, yeah, it's good, it's fine*.” (Kumroj, Interview B5).

As illustrated in the above account, environmental quality was implicitly understood as relating to human well-being, in terms of risks from pollution. Such a location for the community, close to the jungle and away from factories, led to evaluations that the environment was good. Conversely, accounts of pollution from other sources within the community itself, suggested a rather different environmental evaluation. At the same time as some participants positively evaluated the environment being relatively pollution-free, others constructed it as a polluted space due to the way that plastic waste was managed. The problem of plastic waste disposal came up most frequently in Bharatpur:

“…*looking at increasing population, there can be very dangerous pollution. I feel that it will increase, yes, increasing. The use of plastics is increasing and there is no awareness regarding how to maintain cleanliness, how to save us from the problem. They have no such idea. Due to increasing population density, such symptoms are evident*.” (Bharatpur, Interview A14).

Concerns about plastic waste were tied to other concerns about local population increase and the perception that there was a lack of awareness amongst the community in addressing the issue. Such accounts implied that there were no alternatives to using plastic, therefore the problem was in disposing of plastic waste that littered the environment and did not decompose. The main problem causing the pollution was not the presence of plastic waste, but the method used to manage and deal with waste plastic. This chiefly involved collecting the plastic and burning it in open fires. While this resolved the problem of plastic waste littering the community, participants were concerned that the smoke polluted the air and posed risks to health:

“*There is plastic around here and there. To dispose plastic, we need to burn it, and if we burn plastic it makes huge air pollution and affects people's health. The other day I argued with one person not to throw plastic. We must use firewood for cooking and because of that there is again smoke in the air, because of a lack of cooking gas. That's why it has been a very bad environment*.” (Bharatpur; Interview A2).

In contrast to the previous extract constructing the local environment in positive ways as being relatively pollution-free, alternative perspectives such as the above led to very different evaluations of the local environment, with concomitant consequences for the health of the community. While the local community was aware of the contribution of existing informal plastic waste management practices to air pollution, it was nonetheless positioned as being powerless to change in ways that address air pollution as people are locked in to environmentally-damaging practices in order to manage waste and address basic needs. Similar to the need to use firewood for cooking due to shortages of cleaner alternatives, there were no alternatives and burning plastic waste was viewed as unavoidable. Essentially, such accounts lead to a very different evaluation of local environmental conditions.

Conversely, in Kumroj, a municipal system for collecting plastic waste had been in place for some time, therefore the community's method of dealing with plastic waste was not considered to threaten local environmental quality as much as problems such as poor sanitation:

“*People defecate wherever they want around the city area, there are toilets in here no toilets, so people openly defecated. So, I'm concerned about it…Otherwise, there is a facility to collect the waste from municipalities, the van comes and takes away waste. People collect the wastage plastic in sacks, then when the municipality van comes, then they take it away*.” (Kumroj, Interview B6).

The account begins by constructing open defecation as the main issue threatening the environment in nearby Bharatpur, implying a negative assessment of the surroundings. This is contrasted with a more positive assessment where the speaker switches to talk about plastic waste management in Kumroj. Therefore, while plastic waste was a problem in both communities, in evaluating the local environment, the different ways in which plastic waste was managed were used to formulate contrasting assessments of environmental quality overall. These contrasting assessments may also connect to the wider importance of health and well-being, in which potential risks are offset by waste management practices in one community, but raised by plastic waste management practices in another.

#### Deforestation

While plastic waste did not negatively influence environmental assessments in Kumroj as it did in Bharatpur, there were, nonetheless, other issues affecting the community leading to negative environmental evaluations that were not reported in Bharatpur. For people in rural Kumroj, there was a closer felt connection to the neighboring forest as a source of environmental concerns. That is, forest conditions were more commonly invoked in environmental assessments by participants in Kumroj than in Bharatpur. The forest was seen as a valuable community resource, primarily as a source of firewood. Such talk occurred against a context of strikes and fuel shortages, further highlighting the importance of the forest as a source of firewood for local communities, which was being rapidly diminished due to increased demand:

“*We restored the forest with a lot of hard work. The strikes have already led to twenty-five percent of the forest to deforest and if this goes on, the forest will be completely deforested in a year or two. There is a new facility called biogas, we have that facility but, we have seventy-five percent biogas but people are poor and some bring the firewood from the forest, steal it and sell it… People have to survive. Having to die today and struggling for it tomorrow isn't going to work. If you have to survive today, you'll have to work for it today. And if they don't have any other way they'll go to the forest and steal the firewood*.” (Kumroj, Interview B10).

Despite attempts to increase forest cover and reduce reliance on firewood by providing biogas converters within local communities, this did not address the wider problem of sustaining local people's livelihoods, which caused further deforestation and the potential loss of the forest altogether. From this perspective, the amount of forest cover formed an indicator of environmental quality. Furthermore, for participants in Kumroj, the environment was also judged based upon perceived changes in the amount of wildlife that could be observed locally:

“*I think the current environment is worse than the previous environment. I have noticed that the current environment is going down every day instead of going up. Because, previously when I used to go to the jungle I could see the wild animals very near, even sometimes outside of the jungle, but these days we have to go very deep into the jungle to search for the animals*.” (Kumroj, Interview B14).

While the need for wood to sustain people's lives were commonly acknowledged within accounts of the pressures on forest resources in Kumroj, deforestation remained a significant concern.

#### Climate and Weather

While it was not foremost in terms of locally significant issues, participants in Bharatpur and Kumroj also referred to changes in climate and weather conditions in formulating their assessments of the local environment. These changes did not form the basis for positive evaluations of the local environment but appeared in negative or neutral assessments. Talk referred to a narrow range of changes. These mainly involved observations of temperature extremes in which summers were perceived to be increasingly hot, and winters increasingly cold. However, while these observations of climatic change were described causal factors were hardly mentioned. Furthermore, the phenomenon of global climate change was not spontaneously invoked in accounts:

“*I would say it's okay, so far Chitwan's environment is fine, although here is not much forest and plants. In winter it's very cold and summer is getting hotter*.” (Bharatpur, Interview A6).

What appears initially as a positive assessment of the local environment is tempered by a perceived lack of forest cover and greenery in Bharatpur. In addition, the speaker adds the casual observation that winter and summer are increasingly subject to extreme temperatures, though no reason is offered as to why.

In addition to temperature changes, the other way in which the environment was judged was based on fluctuations in precipitation. In such accounts, there was consensus that rainfall was becoming more erratic and less predictable, and that rainfall overall was decreasing, including at the wettest times of the year. Again, no specific reasons were ventured as to why this was happening:

“*Yeah, I think sometimes, I think there's not enough or little rainfall during the rainy season*.” (Bharatpur, Interview A1)

While changes in climate in terms of global averages cannot readily be detected by individuals (Hulme, 2009), participants' observations appeared to reflect general climate trends. However, there was little concern expressed about temperature and precipitation changes, in comparison to other issues linked to health, cleanliness and well-being. Very occasionally, this type of issue was also linked to other perceived environmental problems. For example, one participant associated reductions in the amount of rain that fell to changes in forest cover:

“…*we shouldn't be cutting down trees like we have been doing. We wouldn't get any rain if there weren't any trees*.” (Kumroj, Interview B6).

The above account provides an isolated example of causation in relation to weather related changes. Even so, the role of climate change is not mentioned and rainfall change is attributed solely to the local problem of deforestation. While discussions of weather and climate were almost exclusively focused on the local area, an isolated reference was made to climate change in discussing the environment on a larger scale. One speaker spontaneously referred to broader patterns of global warming observed in changes beyond the local environment:

“…*as you know because of the international global warming, now these days we have maximum cold, maximum hot, and impacts on ice and the change of snow fall trends…now there is very little snow fall in the mountains. If there is snow it melts so fast. These days we can see there are big storms, rainfall, everything has changed now. I think all the weather patterns have changed because of global warming. So, all those things make me concerned about the environment*.” (Kumroj, Interview B13).

While an isolated example, the extract illustrates that climate change did arise in discussions of more local environmental conditions. Broader weather and temperature changes in Nepal corroborated observations at the local level, including temperature extremes and changes in rainfall.

In summary, assessments of the local environment were framed in different ways, leading to differences in the way that environment environmental conditions were evaluated. Assessments were framed based upon locally significant issues, which were both shared by, and individual to each community. Moreover, the most significant concerns were related to health and wellbeing. Next, we consider responses to the question of future environmental change.

### Community Perceptions of Future Environmental Change

Following discussions over present environmental conditions, we then asked participants how the local environment might change in the future. Responses again comprised both positive and negative impressions, with a higher proportion of responses focused on the latter.

**Figure 3:**
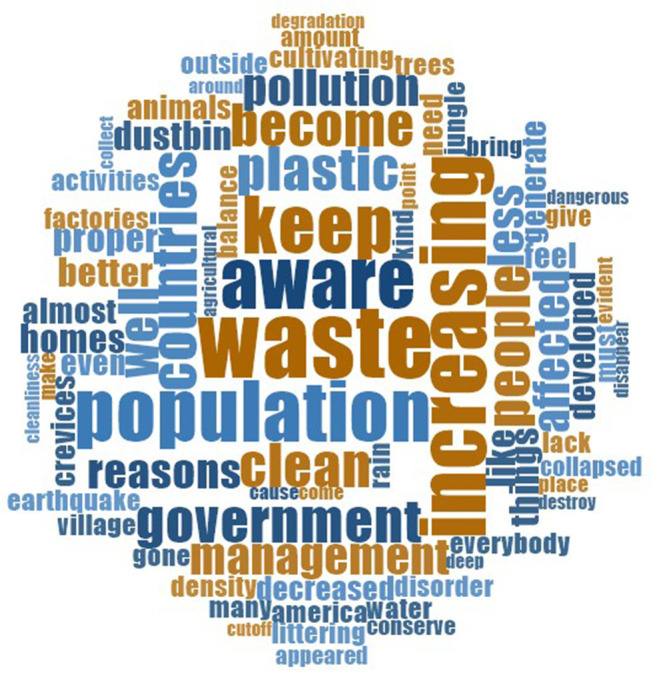
Word cloud of the 75 most common words associated with future environmental conditions (Bharatpur participants).

**Figure 4:**
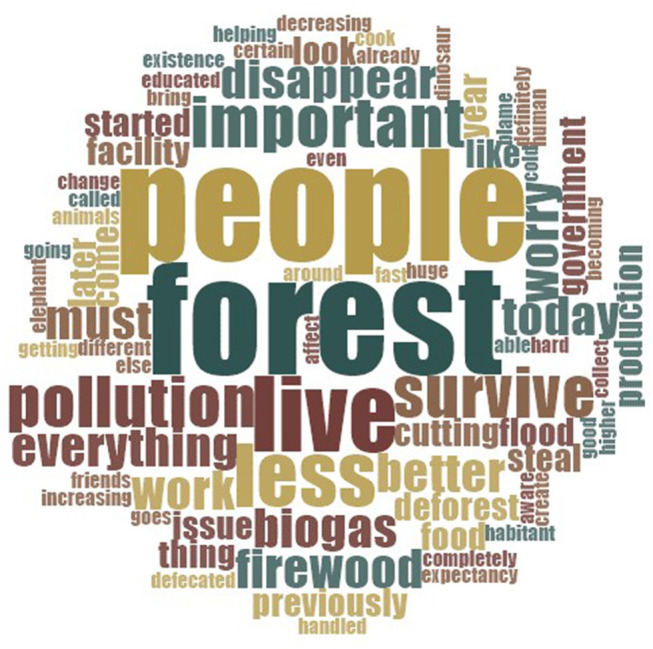
Word cloud of the 75 most common words associated with future environmental conditions (Kumroj participants).

As previously, we created word clouds to get a sense of the sorts of terms that were used in imagining the future, and how these terms varied across communities. The results are shown in Figures 3, 4.

Among participants in Bharatpur, the words “*waste*,” “*population,”* and “*increasing*” came up most frequently in responses about future change. References to negative terms, such as “*pollution*” appeared less than in talk about existing conditions, though it appeared to be used relatively more frequently by participants in Kumroj. With reference to the latter community, the two most prominent words used in talking about the future were “*forest*” and “*people*.” Other terms referred to environmental concerns looking to the future, including “*live*,”, “*less*,” “*important,”* “*survive,”* and “*disappear.”* We now move on to discuss responses in more detail.

#### Future Deforestation

Across both communities, the most commonly reported issue in the future was that of population increase and its consequences, especially for those in Kumroj. As can be read from the analysis so far, population increase influenced environmental perceptions; and was something that was set to continue into the future. Population increase was not viewed in positive ways in either community. Instead, environmental impacts were predicted to increase as more people came to live in the Terai. Of these impacts, the pressure on local forests was most often mentioned. This tapped into the idea that the forest existed as a resource for local communities and that, as a resource, the forest was already being overused:

“*Well, um…I think, the population will increase, they may need more homes, more food, etcetera. For that, the increased population might destroy the green forest for their homes and for cultivating land. There might not be good management of the increased population. There may come disorder in the environment. There might be less wild animals, less trees and plants*.” (Bharatpur, Interview A7).

In addition to providing raw materials in terms of firewood, as mentioned above, the need for land clearance to build settlements and provide food for newcomers compounded deforestation. If not well-managed, there were fears that this would eventually lead to the complete loss of the forest, both as a resource, and as a habitat for local flora and fauna. Such accounts appeared to be grounded in existing concerns about the exploitation of forest resources and served as a warning against continuing unchecked exploitation. In addition to its construction as a community resource and as a habitat for wildlife, in one or two discussions of future population increase, the forest was constructed as a safeguard against preventing other environmental impacts. For example, the forest protected the landscape from flooding and erosion:

“*Since the population and settlements are increasing, the forest is being cut down and people are settling in areas that were forest. More trees are being cut down to meet demand and brick factories are setting up and their chimneys pollute the air with lots of smoke. Because of less forest there could be floods and landslides, so this is the way the environment will be affected in future*.” (Kumroj, Interview B11).

Further to the above, while participants were asked about environmental change in the future, discussions were typically grounded in perceptions of the present. Within the above extract, indications of future conditions linked to increased population and natural disasters are connected with conditions in the here and now, which are projected into an imagined future. It is assumed that present conditions will remain stable and consistent, with little expectation of change. As such, these accounts of the future highlight anxieties linked to present conditions, along with a sense of futility and helplessness that little will change. Conversely, issues such as sanitation did not really come up as future concerns, which likely reflects perceptions of sanitation improvements in the present, compared to the past.

#### Future Temperature Increases and Reduced Precipitation

Of relevance to climate change, rising temperatures, reduced rainfall and the loss of water resources also came up as potential future conditions locally. As found previously in accounts of present conditions, such talk tended to report conditions without elaborating on reasons as to what might contribute to causing them, or by offering opaque references to some unspoken (or non-understood) conditions or circumstances as “*having changed*”:

“*Yes, I think the environment might change. We even hear in the news that the heat or temperature has risen…we also have heard that because of some things the amount of rainfall has also decreased.”* (Bharatpur, Interview A1).

The above narrative hints at climate change, though without any formal acknowledgment of the phenomenon. First of all, the speaker does not refer to direct experience of rising temperatures but formulates this information as something gathered from the media. Likewise, due to a set of unnamed causal factors labeled as “*some things*,” rainfall has also decreased, hinting at complexity. Furthermore, while the speaker begins by stating the belief that the environment could possibly change, the following discussion of climate-relevant change is grounded in changes that have already occurred, rather than changes that could happen in the future. As above, perceptions of future change are intimately connected to changes in the present. This is also confirmed in the next extract, in which a response to the question over future change is also constructed as an account of a present in which the environment locally had shifted from a state of stability to one of flux:

“*When it used to rain in Chure…that is in the mid hills, if we put some grains in the sun to dry then we wouldn't have time to collect them and bring them inside so quickly. The rain would have come, it used to rain quickly. But five to seven years after that there were floods and then other floods, and after that the climate started getting worse and worse. Nowadays what happens is we can see it raining in Chure but here is doesn't rain. So that is a very definite thing that I have noticed*.” (Kumroj, Interview B13).

In this extract, rather than merely hearing about weather and temperature-related changes from secondary sources, evidence of environmental change could be found in the course of changes to practices that were arranged in line with previously stable and consistent weather patterns. As weather patterns had become less predictable, community practices had undergone changes, highlighting the impact of weather-related changes on the local community.

### Local Community Perceptions of Climate Change

The previous sections have shown that while participants in both communities spoke about issues related to changes to temperature and weather, both now in the future, these issues were typically unelaborated beyond the reporting of changes when unelicited, and only rarely connected to wider global climate change. Yet these perceptions often paralleled broader climate change trends. In order to gauge the extent to which participants were aware of climate change, we then asked directly whether participants had heard of climate change or global warming.

**Figure 5:**
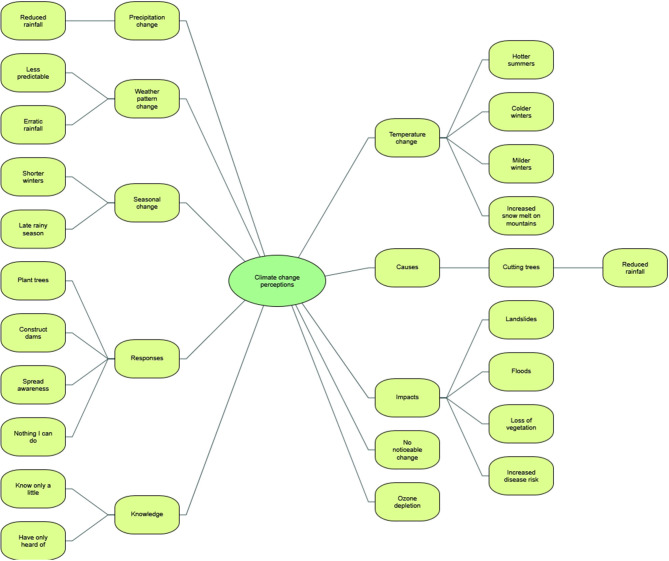
Conceptual map of themes arising in participants' talk about climate change across both communities.

Using NVivo, we began by mapping climate change themes from participants' accounts in both communities., which then formed basic nodes through which to understand the various ways in which participants in both communities talked about climate change. The conceptual map is shown in Figure 5. We then looked at responses in more detail.

#### Changes in Temperature

Of the participants who were directly asked whether they had heard of climate change, only one or two had not, though nobody claimed to know more than a little. Responses were very similar across both communities. Nearly all participants in both communities referred to changes in temperature and/or weather locally. Extreme temperatures were the most commonly cited indicator, most often connected to hotter summers, but also less frequently linked to colder winters, as detailed above in the section on Climate and Weather. Generally, little was said beyond simply noticing change, though one important impact of temperature change in the Terai concerned the direct consequences for plant life:

“*Well…hmm…actually I don't know the reason of global warming. I have heard that the snow of the mountains is melting these days. If it melts it will be hotter. The vegetation will be dry and can't survive, I heard this. It means the temperature increase may affect every living thing on the earth*.” (Bharatpur; Interview A7).

Such talk reflects the importance of agriculture for many communities in what is Nepal's primary agricultural region; while the direct impact on plants was highlighted, other impacts of temperature change were not. The speaker also claims to be unaware of the causes of global warming. However, they construct a link between snowmelt on the distant mountains and temperature rise more locally, with potential impacts for the planet.

Links between climate change and health were rare, however, one speaker explained that hotter temperatures brought new disease risks to humans:

“*What is there is that the rays of the sun, the layer between sun and the earth is what people call depleting nowadays, isn't it? This leads to an increase in heat. This heat has brought about different diseases. Like, mosquito bites cause various diseases. I have heard from the radio that climate change has adversely affected human beings*.” (Bharatpur; Interview A14).

In explaining the causes of temperature rise that bring about health risks from flying insects, the speaker combines elements of ozone layer depletion and global warming. This reflects the way that lay understandings of climate change do not map neatly onto expert definitions, but often overlap with other environmental problems (Rudiak-Gould, 2012).

#### Changes in Precipitation

Following changes in temperature, particularly in the summer months, changes in precipitation were the other main symptom linked to climate change in both communities. An example of this type of perception is provided in Section 3.1.3, though talk of erratic or reduced rainfall was framed locally and very nearly always unconnected with global climate change. However, when the issue of climate change was deliberately elicited by the interviewer, a greater degree of acknowledgment was given to the influence of the phenomenon on local changes particularly in relation to agriculture:

“*Because of global warming, there is not timely rainfall, nothing happens according to the growing seasons. For example, no rain in the rainy reason but it is (rainy) in winter time. Nothing occurring at the right time, I guess this is all the impact of climate change*.” (Kumroj, Interview B13).

Because of global warming, regular patterns of precipitation and the seasons had been thrown into disarray. This was of particular importance in the Terai in terms of agriculture, and was the primary way in which such changes to established patterns were recognized. For others, while erratic weather had recently been observed, it was of little concern as the weather tended not to be consistent but changeable day-to-day:

“*Few years back there was heavy rain, but now there is very little rain, and the summer heat has increased since last year…I think it's not really concerning me because every day is different and going on in its own way, so I don't feel really concerned about it*.” (Kumroj, Interview B12).

In general, accounts of changing temperature and weather were constructed in ways that assumed a transition from the stable and consistent natural patterns of the past, to a present in which established patterns had been disrupted. However, for those less concerned, changes were viewed as part of natural variability. Ultimately, when thinking about local conditions, climate change typically did not form a part of community perspectives unless introduced by the interviewer. The final section summarizes individual climate-relevant behavioral responses to the issues raised in talking about the environment.

#### Health and Well-Being Motivates Engagement in Climate-Relevant Behaviors

Because participants in Bharatpur and Kumroj often did not associate local issues with climate change, there was little talk of the need to adopt specific mitigation or adaptation behaviors. However, within each community one or two climate-relevant behaviors were raised in the course of discussing engagement in more general environmentally-relevant actions. For example, planting trees was widely practiced in both communities. Primarily, this was done to provide wood, create shade around homes and provide fruit. Trees were also considered important in preventing drought (see section on Climate and Weather) and other natural disasters such as flooding and erosion (see section Future Deforestation). In addition, a few participants framed climate-relevant behaviors as motivated by the need to safeguard health and well-being:

“*Trees I plant in the rainy season, so I plant yearly. Once I cut the old, then I plant new…Trees keep the environment clean and healthier. Trees inhale carbon dioxide and exhale oxygen”* (Bharatpur, Interview A6).

While there was no clear link made to climate change, participants acknowledged the value of reducing atmospheric carbon, which was understood as maintaining a “*clean and healthier*” environment. Essentially, such climate-relevant practices were understood not in accordance with received scientific conceptualisations of climate change, but through more pragmatic perspectives linked to health and well-being.

In Kumroj, the Nepalese government had tried to maintain forest stocks by encouraging villagers to purchase biogas converters through grant schemes. Several participants, mainly from Kumroj, had biogas converters. These were seen as advantageous as organic waste could be utilized for producing fuel and then used as a fertilizer. Food could also be cooked quicker without the need to light a fire, and it reduced the need to collect wood. While participants did not mention the link between biogas practices and climate change, one of the most important benefits of biogas was that it did not pollute the air and so reduced health risks linked to inhaling wood smoke:

“*It (biogas) is clean and the air is also clean. The utensils are also not black. Biogas is more hygienic. People can be safe from colds and coughs and smoke-related diseases*.” (Kumroj; Interview B13).

Cleanliness is paramount to the importance of biogas in the above extract. The pollution emitted by burnt wood is illustrated with reference to the condition of cooking utensils, with the implication that the wider environment is being affected in a similar way. In contrast, biogas does not discolor cooking utensils, which illustrates the fuel's superiority in terms of minimizing health risks caused by woodsmoke.

## Discussion

This study set out to investigate community perceptions of environmental and climate-relevant issues within two communities in the Terai region of Nepal. Specifically, we sought to address 4 related research questions; (1) How do community members perceive their environment? (2) How do they see that environment changing in future? (3) To what degree are communities aware of climate change? (4) How important is climate change in comparison to other issues? A range of environmental and climate-relevant issues emerged within current and future perspectives. Perspectives were focused primarily on local issues rather than wider environmental conditions. Issues linked to health and well-being were of paramount importance, while climate change was hardly mentioned in either community, either as a current or future problem. However, there was common awareness of temperature and weather changes in the local climate, though the vast majority did not link these changes to climate change. We now move on to discuss the results of our analyses in more detail.

### Community Perceptions of Current Environmental Conditions

For participants in both communities, assessments of the local environment were commonly based on evaluations of a single locally-salient issue. Positive issues, such as improvements in sanitation over time, invariably resulted in positive overall evaluations of the environment overall. Conversely, pollution resulted in more negative overall assessments. This highlights the highly subjective nature of environmental perceptions and the way in which specific issues can achieve heightened significance in judgements of environmental quality.

Many of the environmental issues raised in both communities were related to health and well-being, including cleanliness, pollution, and deforestation. It may be the relative proximity of each community contributed to this overlapping of issues. It may also be because they represent basic environmental concerns common to many communities—keeping the environment clean, healthy, and pollution free. Similarities between communities may also be partly attributable to our sampling method (see section Study Limitations and Future Research) There were also some differences in issues between communities. While plastic waste was more of an issue in Bharatpur, deforestation came up more often in Kumroj—though neither issue was exclusive to each community.

Climate change as an issue came up only once spontaneously, implying that other local issues were more salient. However, temperature and weather changes analogous to climate change did come up in several interviews across both communities, though without attribution to climate change. There was also little consideration of causal factors beyond immediate local causes such as deforestation affecting precipitation, flooding, and land erosion. In line with Leichenko and Silva (2014), it appeared that temperature and weather changes allied to global climate change were already being experienced, though such issues were more atomized and vernacular and sometimes merged with other environmental problems (Rudiak-Gould, 2012). In line with previous work, community perspectives often drew on different issues without attempting to clearly categorize or explain them (Lorenzoni et al., 2007). Xiao and Dunlap (2007) note how particular environmental cognitions can constrain others; it is therefore possible that, when issues are framed locally, wider frameworks of understanding are overlooked.

### Perceptions of Future Environmental Change

Perceptions of future environmental change were closely linked to mental representations of current conditions and issues of anxiety and concern. This could be seen in the way that participants rarely spoke about sanitation with reference to environmental change in the future, as sanitation had improved within communities. However, concerns about issues linked to current population increase were projected into the future and anticipated to continue unabated. Previous work has found that existing perceptions of self and other can be elicited through projections of “possible selves” in the future (Harrison, 2018). In the same way, communities' imagined environmental futures highlight salient issues within existing relationships between communities and their physical surroundings.

The Terai region has witnessed large increases in population over recent decades (Population Reference Bureau, 2002), and this was linked to pollution, deforestation and pressure on natural resources. While predictions of temperature and weather emerged from the interviews, such impacts were less frequently mentioned than concerns over population growth, as found in other research (e.g., Butler et al., 2014). Before communities can interpret and respond to climate-relevant issues, it may therefore be necessary to address existing concerns characterized by visions of the present and the future. In addition, the analysis highlights the relevance of sociocultural arrangements and cultural practices that contribute to community perspectives. For example, tree-felling was understood sympathetically within wider contexts of survival and economic struggle, as well as fuel shortages that left no alternative but to take wood from the forest. Such perspectives serve to highlight the complex nature and wider structural relations sustaining environmentally damaging practices.

### Awareness of Climate Change

Climate awareness was relatively unmentioned in discussing the local environment, echoing previous studies (Gallup, 2009; Withana and Auch, 2014). We found little difference between awareness in Bharatpur and Kumroj. A potential reason for this is that the changes observed suggest broader shifts in temperature and weather affecting the wider region, rather than localized effects or micro-climates that might affect one community and not another. Other studies have also reported lower awareness in rural communities (Tanner et al., 2018), though a lack of difference may be due to the higher levels of environmental awareness from NGO engagement in Kumroj. However, while most participants did not spontaneously discuss the issue of climate change, when directly questioned, all had at least heard of climate change and many were able to eloquently demonstrate a good degree of knowledge. Therefore, it may not have been that participants were unaware of climate change, but simply did not consider it a locally salient issue. Tanner et al. (2018) also found that climate change awareness was low, despite respondents observing changes to local weather and climate. It may be that communities look to more local explanations for climate-relevant issues, as was found in some discussions. Therefore, if received knowledge teaches that the lack of rain is due to local forests being depleted, why would communities look to wider, more nebulous phenomena as explanations? The kinds of issues that came up in talk of climate change broadly reflects other research on community perceptions of climate change in the Terai (e.g., Tiwari et al., 2010; Maharjan et al., 2011). The apparent disjuncture between local experience and climate change suggests that the latter may lack relevance for local communities as long as environmental changes can be attributed to more local causal factors. It also suggests two kinds of climate change; a distanced, abstract climate change, and a more experiential, locally-grounded one. Within communities facing such impacts there is a need for a nuanced understanding that blends both. Howe et al. (2013) remark that local perceptions, such as temperature change, can positively bias perceptions of more abstract global climate change, which in turn can generate greater awareness and the capacity to respond to reduce risks to communities. As communities appear to be aware that the local climate is changing in a variety of ways, it is necessary to translate this awareness beyond the local. Reciprocally, more global perspectives need to connect with the concerns and interests of communities at the local level to make climate change more relevant to people's everyday lives. Bain et al. (2016) discuss evidence for initiatives promoting public engagement designed to generate support on the basis of considerations that are independent of climate change, including health and the creation of benevolent communities.

### Study Limitations and Future Research

The use of a single qualitative methodological approach utilizing a small sample can only provide a partial insight into climate-relevant and environmental issues confronting the communities studied. Qualitative interview methods rely heavily on participants being able to recall and clearly convey their thoughts in the limited context of the interview interaction. Managing interview interactions in a cross-cultural setting remains a significant challenge and it is possible that the framing of questions could have influenced responses, such as precluding the discussion of global climate change by not deliberately eliciting the topic early in the interviews. Triangulation using other methods and larger samples might help to clarify these qualitative findings. Convergence in perspectives between communities may be attributable to our sampling method. While we categorized Bharatpur as the urban counterpart to rural Kumroj, most participants lived on the edges of the city close to the countryside, which may have generated perceptions that were more aligned with a rural, rather than an urban perspective. Future research might further investigate the apparent disparity between awareness of climate change more generally, and a lack of acknowledgment of climate change in discussions of environmental conditions at the local level. Drawing attention to this gap might also serve to elicit more comprehensive community perspectives and rule out potential shortcomings of a single methodological approach.

## Data Availability

The raw data supporting the conclusions of this manuscript will be made available by the authors, without undue reservation, to any qualified researcher.

## Ethics Statement

This study was carried out in accordance with the recommendations of the Ethics Policy, Cardiff University School of Psychology. The protocol was approved by the Cardiff University School of Psychology Ethics Committee. All subjects gave written informed consent in accordance with the Declaration of Helsinki.

## Author Contributions

NN, IC, and RM conducted fieldwork with the assistance and guidance of LW and SC. NN was primarily responsible for analysis and authorship of the paper, with significant contributions in both areas from the other authors. All authors agree to be accountable for the content of the work.

### Conflict of Interest Statement

The authors declare that the research was conducted in the absence of any commercial or financial relationships that could be construed as a potential conflict of interest.
