# Supplementary material for: Perceptions of Local Environmental Issues and the Relevance of Climate Change in Nepal's Terai: Perspectives From Two Communities
Source: Front Sociol. 2019 Aug 20;4:60. doi: 10.3389/fsoc.2019.00060 (PMC8022627; doi:10.3389/fsoc.2019.00060)
Supplement: Supplementary file 1 [file Table_1.docx]

**Interview questions on which the analysis is based**

**Environmental and climate-relevant perspectives:**

1. Can you tell me what the environment means to you?
2. How important are environmental issues/values to you in the context of your life?
3. What is the environment like here?
4. Do you think that the environment will change in the future? If so, how?
5. Have you heard of climate change or global warming? If so, what have you heard?
